# Development of a Diagnostic IgM Antibody Capture ELISA for Detection of Anti-Cache Valley Virus Human IgM

**DOI:** 10.4269/ajtmh.24-0360

**Published:** 2024-11-19

**Authors:** Christin Goodman, Jordan A. Powers, Sierra R. Mikula, Holly R. Hughes, Brad J. Biggerstaff, Kelly Fitzpatrick, Amanda J. Panella, Carlos Machain-Williams, SeungHwan Lee, Amanda E. Calvert

**Affiliations:** ^1^ Division of Vector-Borne Diseases, U.S. Centers for Disease Control and Prevention, Fort Collins, Colorado;; ^2^ Unidad Profesional Interdisciplinaria de Ingeniería Palenque (UPIIP), Instituto Politécnico Nacional, Palenque, Mexico;; ^3^ Department of Clinical Pharmacology and Therapeutics, Seoul National University, Seoul, South Korea

## Abstract

Cache Valley virus (CVV), a mosquito-borne orthobunyavirus, causes epizootics in ruminants characterized by congenital malformations and fetal death in North America. Only seven human infections have been identified; limited information exists on its potential as a human teratogen. Diagnosis of CVV infections relies on the plaque reduction neutralization test (PRNT), which requires live virus, is time-consuming, and cannot differentiate between recent and past infections. To improve diagnostics for CVV, we developed an IgM antibody capture ELISA (MAC-ELISA) for detection of anti-CVV human IgM in diagnostic specimens that can be performed faster than PRNT and is specific to IgM, which is essential to determine the timing of infection. Conjointly, a cell line constitutively expressing human-murine chimeric antibody with the variable regions of monoclonal antibody CVV-17 and constant regions of human IgM was developed to provide positive control material. The new cell line produced antibody with reactivity in the assay equivalent to that of a human serum sample positive for anti-CVV IgM. Five of seven archived human specimens diagnostically confirmed as CVV positive tested positive in the MAC-ELISA, whereas 44 specimens confirmed positive for another arboviral infection tested negative, showing good initial correlation of the CVV MAC-ELISA. Two of 27 previously collected serum samples from febrile patients in Yucatán, Mexico, who tested negative for a recent flaviviral or alphaviral infection were positive in both the MAC-ELISA and PRNT, indicating a possible recent infection with CVV or related orthobunyavirus. The MAC-ELISA described here will aid in making diagnostics more widely available for CVV in public health laboratories.

## INTRODUCTION

Cache Valley virus (CVV) is a mosquito-borne virus in the genus *Orthobunyavirus*, family *Peribunyaviridae*, that was first isolated from a *Culiseta (Cs.) inornata* mosquito pool in Cache Valley, Utah, in 1956.^1^ It has since been shown to have a wide geographic distribution across North America.^2^^,^^3^ Cache Valley virus is classified in the Bunyamwera serogroup, whose other members include Tensaw, Maguari, Northway, Potosi, Main Drain, Fort Sherman, and Lokern viruses.^4^ Cache Valley virus has been isolated from a number of different mosquito species, including *Anopheles (An.) punctipennis* (Say), *Anopheles quadrimaculatus* (Say), *Aedes (Ae.) sollicitans* (Walker), *Coquillettidia perturbans* (Walker), *Cs. inornata* (Williston), *Aedes trivittatus* (Coquillett), and *Aedes canadensis* (Theobald), but principal vectors remain undetermined. White-tailed deer are thought to be the main amplifying hosts, although little research has been done in this area.^5^

Orthobunyaviruses have a three-segmented negative-sense single-stranded RNA genome that encodes three structural proteins, the nucleocapsid (N) and two glycoproteins (Gn and Gc). The N protein is approximately 26 kDa in size and encapsidates the segments of the genome and several copies of the viral RNA-dependent RNA polymerase. A lipid bilayer surrounds the nucleocapsid, with Gc (110 kDa) and Gn (32 kDa) displayed as a lattice of heterodimer spikes on the surface of the virion.^6^ The nucleocapsid has been shown to be involved in viral replication, whereas the glycoproteins engage in receptor-mediated endocytosis and virus-cell membrane fusion.^6^

Cache Valley virus can cause severe epizootics among sheep flocks resulting in stillbirths and fetal abnormalities with defects in the central nervous and musculoskeletal systems of affected lambs. Seroprevalence in sheep is high, with 28% and 64.6% documented in the United States and Saskatchewan, Canada, respectively. Anti-CVV neutralizing antibodies have also been documented in horses, cattle, and other domestic animals.^7^^,^^8^

Seroprevalence of CVV in humans has been documented as low as 3% and as high as 18%.^9^^–^^11^ Only seven clinical cases of CVV disease have been identified, and three of these were fatal.^12^^–^^18^ A link to CVV infection in humans and congenital malformations or neural defects has not been identified, and only two studies have investigated this question. A serologic study conducted retrospectively from specimens collected from women postpartum (*N* = 500) in the early 1960s showed a correlation between mothers with anti-CVV antibodies who had given birth to newborns with macrocephaly.^19^^,^^20^ However, this study could not determine the timing of infection, because specific anti-CVV IgM present during the acute and early convalescent phases of the infection was not identified. The other study determined that CVV infection was not associated with a rise in infants born with anencephaly in south Texas between 1990 and 1991, as none of the mothers had anti-CVV neutralizing antibody.^20^

Given that CVV is understudied and infection is potentially underrecognized because of a lack of serology assays available, CVV is not routinely included in diagnostic testing of suspected arboviral disease cases. There are no commercial tests or kits available for the diagnosis of CVV in humans or animals. Laboratory-developed molecular assays such as reverse transcription polymerase chain reaction are available but are not infallible and may miss CVV cases in some instances because of the transient nature of the viremia. Serological diagnosis is limited to the use of the plaque reduction neutralization test (PRNT), which requires work with live virus in cell culture, takes several days to run, and cannot indicate the timing of infection because the antibody isotype is not differentiated. Rapid immunoassays, such as ELISA or multiplex immunoassay for the detection of IgM and IgG, are faster, eliminate the need for cell culture, and can help indicate the timing of infection based on the antibody isotype detected in the assay.

To meet the need for a faster serologic assay for the detection of anti-CVV IgM in human specimens, we developed an IgM antibody capture (MAC)-ELISA. A murine-human chimeric IgM antibody expressing variable regions of a murine monoclonal antibody (MAb) on the human IgM backbone was expressed constitutively in human embryonic kidney-293 (HEK-293) cells and developed for use as a positive control. In this report, we describe the evaluation of the CVV MAC-ELISA in conjunction with the surrogate engineered control and its performance relative to that of CVV antibody-positive and -negative human specimens. The CVV MAC-ELISA was also used to evaluate samples from patients in Yucatán, Mexico, who may have had previous exposure to CVV.

## MATERIALS AND METHODS

### Viruses, cells, and diagnostic specimens.

Cache Valley virus strain W1-03BS7669 (lineage 1) was obtained from the Arboviral Diseases Branch, Reference and Reagent Laboratory (Fort Collins, CO). Cache Valley virus stock was grown in African green monkey kidney (Vero) cells to a titer of 5.0 × 10^7^ plaque-forming units (PFU) per mL. Vero cells, baby hamster kidney (BHK-21) clone 15 cells, rhesus monkey kidney (LLC-MK2) cells, and Vero clone E6 cells were maintained in Dulbecco’s modified Eagle medium (catalog no. 11965118; Invitrogen, Waltham, MA) with 10% fetal calf serum (FCS) (catalog no. 1500-500G; VWR, Radnor, PA), 2 mM L-glutamine (catalog no. 25030-081; Invitrogen), 0.15% sodium bicarbonate catalog no. 25080-094; Gibco, Waltham, MA), 10,000 U/mL penicillin-streptomycin (catalog no. 15140-122; Gibco) at 37°C in 5% CO_2_. HEK-293 cells (HEK-293.2sus CRL-1573.3; ATCC, Manassas, VA) were grown in a 1:1 mixture of Expi-293 medium (ThermoFisher, Waltham, MA) and Ex-Cell medium (Millipore Sigma, Burlington, MA) supplemented with 1% (vol/vol) penicillin-streptomycin (ThermoFisher Scientific). Cells were incubated at 37°C with 5% CO_2_ in suspension with constant mixing at 130 rpm on orbital shaking platforms (ThermoFisher).

Ethical approval for the use of archived human diagnostic specimens was obtained from the CDC’s Human Subjects Institutional Review Board (CDC IRB no. 6773). Ethics approval for the collection and testing of serum samples from patients presenting to medical clinics in Yucatán, Mexico, with a suspected viral infection who tested negative in molecular assays for flavivirus and alphavirus infections, as well as enteroviruses, was obtained from a registered SISTPROY project (CIRB-2021-0011) at the Regional Research Center “Dr. Hideyo Noguchi,” Universidad Autónoma de Yucatán, The Korea Friendship Hospital Research Committee (HCM/ENS/51/07/2021), and Research Ethics Committee of the Guerrero State Health Services Registry (#CEISSG 001140519).^21^^–^^23^

### Plaque reduction neutralization test.

To assess neutralization activity of archived diagnostic specimens, samples were tested according to the routine CDC diagnostic PRNT standard operating procedure. Briefly, serum samples were heat inactivated at 56°C for 30 min. Single replicate samples were diluted 1:5, serially titrated 2-fold, and then mixed with equal volumes of CVV strain 6V633 at a concentration of 200 PFU/0.1 mL in BA-1 (Hanks M-199 salts [catalog no. M9163; Sigma-Aldrich, St. Louis, MO], 0.05 M Tris pH 7.6 [catalog no. 15567027; Gibco], 1% bovine serum albumin [catalog no. 81-066-4; Millipore, Burlington, MA], 0.35 g/L sodium bicarbonate [catalog no. 25080–094; Gibco], 10,000 U/mL penicillin-streptomycin [catalog no. 15140–122, Gibco], 1 mg/L Fungizone [catalog no. SV30078.01; HyClone, Marlborough, MA]) with 8% normal human serum (NHS) containing exogenous complement. Serum-virus mixtures were incubated for 1 hour at 37°C with 5% CO_2_, and 0.1 mL of each mixture was then inoculated onto Vero cells in six-well plates and incubated for 1 hour at 37°C with 5% CO_2_. A 0.5% agarose-nutrient medium overlay (SeaKem LE agarose [catalog no. 50004; Lonza, Rockland, ME], 0.165% lactalbumin hydrolysate [catalog no. 259962; VWR], 0.033% yeast extract [catalog no. 212750; ThermoFisher Scientific), Earle’s balanced salt solution, and 2% fetal bovine serum) was applied, and plates were incubated at 37°C with 5% CO_2_ for 3 days, during which a second 0.5% agarose-nutrient medium overlay containing a neutral red stain (catalog no. 091691149; MP Biomedicals, Santa Ana, CA) was applied.^24^ Plaques were counted on day 4. Positive and negative controls, along with back-titrations, were run in duplicate and used for assay validation. Neutralizing antibody titers were calculated as the reciprocal of the highest serum dilution that reduced the CVV plaque count by 90% based on back-titrations. Results were interpreted as follows: PRNT titer ≥10 = positive, PRNT titer <10 = negative.

To assess neutralization activity of serum samples from archived diagnostic specimens from patients residing in Yucatán, Mexico, samples were tested in duplicate in PRNT as described above, but with the following exceptions. Approximately 50 PFU of CVV strain W1-03BS7669 was incubated with equal amounts of serial 2-fold dilutions of sera starting at a 1:20 dilution, mixed, and incubated for 1 hour at 37°C with 5% CO_2_, before virus-antibody mixtures were inoculated onto Vero cells in 12-well plates. The second overlay containing neutral red stain was added 48 hours after incubation. Plaques were counted 3 days after infection, and percent neutralization was calculated based on input virus titer. Virus neutralization curves were generated by a four-parameter logistic curve regression dose response used to calculate each sample’s 90% inhibitory concentration (IC_90_) values using GraphPad Prism v.6 with the bottom and top of the curves constrained to 0 and 100, respectively.

### Viral antigen production and inactivation.

Cache Valley virus strain WI-03BS7669 was inoculated into T150 cm^2^ flasks individually seeded with Vero, Vero clone E6, BHK-21 clone 15, and LLC-MK2 cells at a multiplicity of infection (MOI) of 0.01. Flasks were incubated at 37°C with 5% CO_2_ for 3 days. On day 3, cell culture supernatant was harvested, clarified at 2,400 ×* g* for 10 min at 4°C, and stored at −70°C with 20% FCS until further analysis. Cell culture supernatants were then thawed, and approximately 10 mL of supernatant was treated with beta-propiolactone (BPL; catalog no. W91701; CTC Organics, Atlanta, GA) at a final concentration of 0.1% and incubated for 48 hours at 4°C with moderate shaking on a refrigerated shaker plate. Due to acidic BPL by-products, 7.5% sodium bicarbonate (catalog no. 25080-094; Gibco) was added intermittently to adjust the pH.^25^ Postincubation, BPL-treated samples were concentrated approximately 10-fold in Amicon Ultra-15 100-kDa centrifugal filter devices (catalog no. UFC910024; Millipore Sigma) at 3,500 ×* g* for 10 to 15 min at 18°C and stored at −70°C until further analysis.

### Antibody sequencing and analysis.

RNA from hybridoma cells was extracted using the RNeasy kit (catalog no. 74004; Qiagen, Germantown, MD), and messenger RNA (mRNA) was selected using the RNeasy Pure mRNA bead kit (catalog no. 180244; Qiagen). Sequencing analysis from libraries of complementary DNA (cDNA) was performed as previously described.^26^ Briefly, sequencing was performed on the Ion GeneStudio S5 (ThermoFisher Scientific) with Ion 520 chips prepared on the Ion Chef instrument with the Ion 510, 520, and 530 Chef kit (catalog no. A34461; ThermoFisher Scientific). De novo assembly of fastq sequences was done in CLC Genomics Workbench v. 22 (Qiagen), contigs were analyzed with NCBI BLAST analysis (www.ncbi.nlm.nih.gov), a final reference guided assembly in SeqMan NGen v. 15 (DNAStar, Madison, WI), and heavy and light chain variable sequences were identified using IMGT/V Quest alignment (www.igmt.org).

### Cloning and Gibson assembly of plasmid expressing murine-human chimeric IgM.

Primers for amplification and cloning (Supplemental Table 1) were designed to be 40 nucleotides in length, with 20 nucleotides complementary to the antibody’s signal sequence and 20 nucleotides complementary to the vector. Antibody variable region and vector fragments for assembly were amplified using the Q5 high-fidelity 2× master mix kit (catalog no. M0492; New England Biolabs, Ipswich, MA) in accordance with the manufacturer’s instructions and assembled using the NEBuilder HiFi DNA assembly reaction (catalog no. E5520; New England, Biolabs) in accordance with the manufacturer’s instructions. Plasmids were extracted from transformed chemically competent *Escherichia coli* DH5α cells using a QIAprep spin miniprep kit (catalog no. 27106X4; Qiagen), and heavy and light chain inserts were verified by Sanger sequencing on an ABI 3130 instrument (Applied Biosystems, Waltham, MA).

### Development of HEK-293 cells constitutively expressing murine-human chimeric antibody.

HEK-293 cells constitutively expressing the CVV-17 murine-human chimeric IgM (CVVhIgM17) were made essentially as previously described.^26^ Briefly, HEK-293 cells were electroporated with CVVhIgM17 expression vector described above. Cells were allowed to recover overnight before an immunofluorescence assay (IFA) was performed to verify antibody expression using a goat anti-human human IgM Fc_5µ_ antibody conjugated to fluorescein (catalog no. 109-095-043; Jackson Immunoresearch, West Grove, PA). Transfected HEK-293 cells were seeded into medium containing 3% carboxymethyl cellulose (catalog no. C4888; Millipore Sigma) in growth medium and grown for 7–14 days before colonies were picked and placed into 96-well plates (Corning, Tewksbury, MA) containing growth medium. Clones were grown for 7–10 days before screening with IFA to identify clones with at least 50% cells expressing human IgM. Stably expressing cells were selected based on expression of antibody using IFA. Clones with ≥95% expression of antibody in IFA were expanded, and supernatant was collected for antibody purification.

Clonal stability was determined by passaging cells 10 times and testing cell culture supernatants in MAC-ELISA as previously described.^26^ Regression spline fits of expression (positive/negative (P/N) value calculated as the mean OD450 value of the CVV-positive human reference serum, CVVhIgM17, or archived serum sample reacted on CVV antigen (P), divided by the mean OD450 value of the negative human reference serum reacted on CVV antigen (N)) against log dilution was fitted to all passages, and a mean curve was estimated using methods previously described.^27^ The endpoint titer (EPT) was estimated from the spline curves for each passage as the lowest dilution that resulted in a positive (P/N) value of 3. The EPTs of each passage were analyzed using least absolute deviation (LAD) regression to evaluate the slope of the regression line of the EPT passage sequence number using the Wald test. Analyses were performed using the R software package (www.r-project.org).

### Murine-human chimeric IgM antibody purification.

Chimeric IgM antibodies were purified using the Pierce IgM purification kit (catalog no. 44897; ThermoFisher Scientific) according to the manufacturer’s instructions. Protein concentrations were determined by the Bradford assay (catalog no. 5000006; Bio-Rad, Hercules, CA) according to the manufacturer’s instructions.

### IgM antibody capture ELISA.

A MAC-ELISA was developed for the detection of anti-CVV human IgM, as previously described.^28^ Briefly, human reference sera, either positive or negative for CVV IgM, were used as positive or negative controls, respectively; additionally, positive human serum (PHS) for high (PHS-1) or low (PHS-2) levels of CVV neutralizing antibody were used during assay optimization and archived sample testing and were diluted 1:400 (or 1:1,000 during the CVVhIgM17 titration). Concentrated cell culture supernatant from HEK-293 cells expressing CVVhIgM17, engineered and produced as a replacement CVV IgM control, was diluted 1:3,000 unless evaluated as part of a serial titration. Inactivated CVV Vero cell culture supernatant was used as virus-specific antigen, and normal Vero cell culture supernatant was used as normal antigen; antigens were diluted 1:200 unless evaluated as part of a serial titration. Monoclonal antibody CVV-14 was conjugated to horseradish peroxidase (HRP) and diluted 1:4,000 (or 1:8,000 during the antigen titration), unless evaluated as part of a serial 2-fold titration. Archived serum samples were tested at 1:400. The MAC-ELISA results were expressed as a P/N value described above. When applicable, nonspecific background reactivity (NBR) was assessed and defined as the mean OD450 value of the CVV-positive human reference serum, CVVhIgM17, or archived serum sample reacted on CVV antigen, divided by the mean OD450 value of the same corresponding sample reacted on normal antigen. Results were calculated as follows: positive, P/N ≥3.0 and NBR ≥2.0; equivocal, 2.0 ≤ P/N < 3.0 and NBR ≥2.0; negative, P/N <2.0 and NBR any value; uninterpretable, P/N ≥2.0 and NBR <2.0.

## RESULTS

### Reagent preparation and optimization for MAC-ELISA.

**Figure 1.**
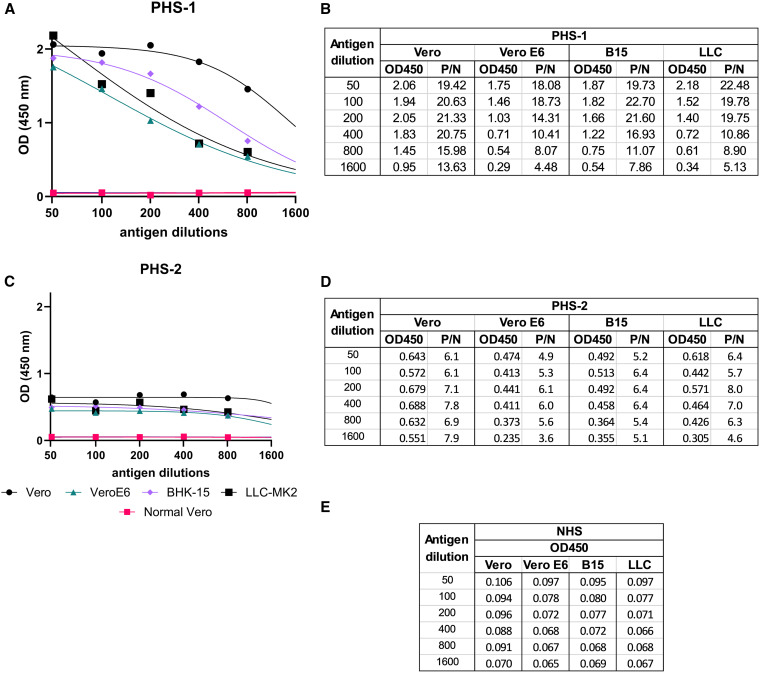
Evaluation of Cache Valley virus (CVV) antigen produced in tissue culture in IgM antibody capture (MAC)-ELISA. Cache Valley virus antigen was made by infecting Vero, Vero E6, BHK21c.15 (B15), and LLC-MK2 (LLC) cells, inactivating viral cell culture supernatant with 0.1% beta-propiolactone, and concentrating inactivated supernatant approximately 10-fold. Antigen was titrated in the MAC-ELISA to observe optical density (OD) reactivity when tested using positive human serum (PHS) samples with high (**A**) and low (**C**) titers of CVV neutralizing antibody. P/N values (defined as the mean OD450 value of the serum sample reacted on CVV antigen divided by the mean OD450 value of normal human serum [NHS] [**E**] reacted on CVV antigen) of the human serum samples with high (**B**) and low (**D**) titers of CVV neutralizing antibody were calculated.

Cache Valley virus antigen was produced from infected tissue culture in Vero, Vero E6, BHK-21c.15, and LLC-MK2 cells. Cells were infected at an MOI of 0.01, and cell culture supernatant was harvested 3 days later. After inactivation of virus with 0.1% BPL and concentration approximately 10-fold, antigen was titrated in the MAC-ELISA from 1:50 to 1:1,600 dilutions to evaluate OD reactivity when tested using CVV-positive human serum samples with high (Figure 1A) and low (Figure 1C) titers of CVV-neutralizing antibody. When tested with the high CVV neutralizing antibody serum sample (PHS-1) and assessing P/N values at the higher antigen dilutions of ≥1:400 to account for the prozone effect, CVV antigen produced in Vero cells gave the highest values overall in comparison with CVV antigen produced in Vero E6, BHK-21c.15, and LLC-MK2 cells at the same respective dilutions (Figure 1B). When tested with the low CVV neutralizing antibody serum sample (PHS-2) and assessing P/N values at the higher antigen dilutions of ≥1:400 to account for the prozone effect, CVV antigen produced in Vero cells again gave the highest values overall in comparison with CVV antigen produced in Vero E6, BHK-21c.15, and LLC-MK2 cells at the same respective dilutions (Figure 1D). A 1:200 dilution of CVV antigen produced in Vero cells was selected for use in all subsequent assays due to the start of the CVV Vero antigen titration in the linear range on PHS-1.

**Figure 2.**
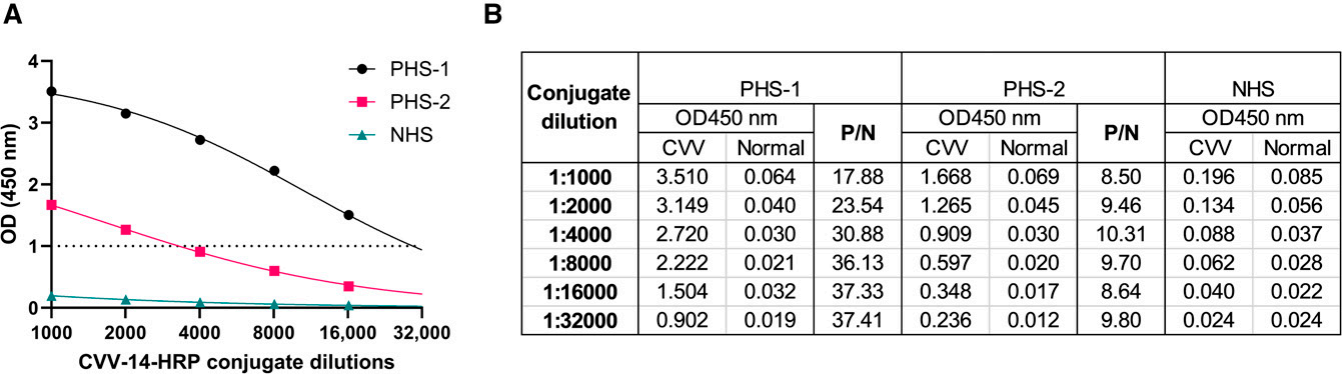
Evaluation of the performance of horseradish peroxidase (HRP)-conjugated anti-CVV-14 as detector antibody in IgM antibody capture (MAC)-ELISA. (**A**) Anti-CVV-14 was conjugated to HRP and titrated in the MAC-ELISA to observe optical density (OD) reactivity when tested using positive human serum (PHS) samples with high (PHS-1) and low (PHS-2) titers of CVV neutralizing antibody and normal human serum (NHS). (**B**) P/N values (defined as the mean OD450 value of PHS reacted on CVV antigen divided by the mean OD450 value of NHS reacted on CVV antigen) were calculated. CVV = CVV Vero cell antigen; Normal = uninfected Vero cell antigen.

Anti-CVV MAb CVV-14 was purified and conjugated to HRP for use as the detector antibody in the MAC-ELISA.^29^ CVV-14–HRP was titrated from 1:1,000 to 1:32,000 in the MAC-ELISA and evaluated for OD reactivity when tested using CVV-positive human serum samples with high (PHS-1) and low (PHS-2) titers of CVV-neutralizing antibody and NHS (Figure 2A). Optical densities close to 1.0 were used to determine the optimal dilution of the conjugate in the assay. When reacted with PHS-1, an OD of 0.902 was recorded at a 1:32,000 dilution of the conjugate, with a calculated P/N value of 37.41 (Figure 2B). When reacted with PHS-2, an OD of 0.909 was recorded at a 1:4,000 dilution of the conjugate, with a calculated P/N value of 10.31 (Figure 2B). A 1:4,000 dilution of CVV-14–HRP conjugate was selected as the dilution for all subsequent assays because both positive human sera had ODs ≥0.90 in the assay.

### Design, construction, and expression of CVV murine-human IgM chimeric antibody.

Human-murine IgM constructs for use as a positive control in the MAC-ELISA were engineered using variable regions of MAbs as previously described.^29^ Total RNA was extracted from hybridoma cells, and mRNA was enriched and sequenced by next-generation sequencing. Variable region sequences were determined using IMGT/V, and primers were designed to amplify the variable regions starting at the signal sequence through framework region 4 (Supplemental Table 1). Amplified heavy variable (V_H_) and kappa variable (V_ĸ_) regions replaced the variable regions of the Trastuzumab-IgM/ĸ in the pVITRO plasmid, and HEK-293 cells were transiently transfected with each plasmid.

### Anti-CVV human-murine chimeric IgM performance in CVV MAC-ELISA.

**Figure 3.**
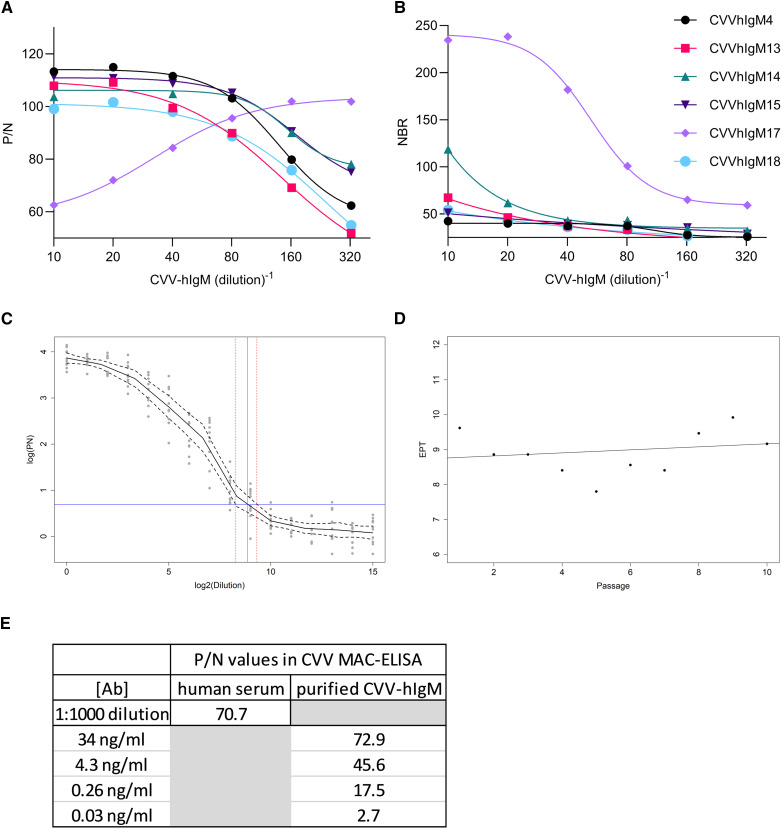
Selection and performance of Cache Valley virus human IgM antibody (CVV-hIgM) from a recombinant cell line. (**A**) P/N values (mean value of optical density at 450 nm [OD450] of the CVV-hIgM reacted on CVV antigen divided by the mean OD450 value of normal human serum [NHS] reacted on CVV antigen) of supernatant from transiently transfected HEK-293 cells expressing CVV-hIgM with variable regions from murine monoclonal antibodies (MAbs) CVV-4, CVV-13, CVV-14, CVV-15, CVV-17, and CVV-18 diluted 1:10 to 1:320 in the CVV IgM antibody capture (MAC)-ELISA. (**B**) Nonspecific background reactivities (NBRs) (mean OD450 value of the CVV-hIgM reacted on CVV antigen divided by the mean OD450 value of CVV-hIgM reacted on Vero normal antigen) of supernatant from transiently transfected HEK-293 cells expressing CVV-hIgM with variable regions from murine MAbs as described for panel A. (**C**) Predicted mean values of CVVhIgM17 endpoint titer (EPT) (black line) from the cell line were plotted as log (P/N) versus log_2_(dilution), with confidence bands shown as dashed black lines. The reference line in blue for determining the EPT is shown as log_2_. The estimated EPT (95% CI) is indicated with vertical red lines with confidence bands as dashed red lines. (**D**) EPT against passage number evaluated by least absolute deviation (LAD) regression test (black line) shows stable secretion of CVVhIgM17 from the cell line tested by MAC-ELISA. (**E**) Purified CVVhIgM17 was titrated in the MAC-ELISA with results expressed as a P/N value.

Six anti-CVV human-murine IgM chimeric antibodies based on CVV murine MAbs CVV-4, CVV-13, CVV-14, CVV-15, CVV-17, and CVV-18 and transiently expressed in HEK-293 cells were evaluated in the CVV MAC-ELISA to determine the optimal construct for cell line development. All CVV-hIgM constructs reacted positively with a P/N value >3.0 at all dilutions tested (a presumptive positive in the diagnostic algorithm) (Figure 3A). Human-murine chimeric IgM constructs CVVhIgM4, CVVhIgM13, CVVhIgM14, CVVhIgM15, and CVVhIgM18 reacted similarly in the MAC-ELISA with P/Ns at the lower dilution of 1:10 between 99 and 113 (CVVhIgM4 = 113, CVVhIgM13 = 108, CVVhIgM14 = 104, CVVhIgM15 = 111, CVVhIgM18 = 99) and P/Ns at the highest dilution of 1:320 between 52 and 78 (CVVhIgM4 = 62, CVVhIgM13 = 52, CVVhIgM14 = 78, CVVhIgM15 = 75, CVVhIgM18 = 55) (Figure 3A). While CVVhIgM17 had the lowest reactivity in the lower dilutions of supernatant, the P/Ns increased with increasing dilutions of supernatant, indicating a prozone effect demonstrating an excess of antibody in the assay (at a 1:10 dilution, P/N = 63, at a 1:320 dilution, P/N = 102) (Figure 3A). To determine the level of antibody nonspecifically binding in the assay, NBRs of the human-murine chimeric IgM constructs were determined using the ratios of the OD450 absorbance of the antibody on viral antigen to the OD450 absorbance of the antibody on Vero normal antigen (Figure 3B). All constructs displayed little nonspecific reactivity in the assay with NBR ratios >3.0 for all antigens tested when titrated to 1:320 (CVVhIgM4 = 26, CVVhIgM13 = 21, CVVhIgM14 = 32, CVVhIgM15 = 29, CVVhIgM18 = 24) (Figure 3B). CVVhIgM17 had the highest NBR ratios at 235 when tested at a 1:10 dilution and 59 when tested at a 1:320 dilution (Figure 3B). CVVhIgM17 was ultimately selected as the final construct because it yielded the highest P/N and NBR values at the highest antibody dilution of 1:320.

A cell line expressing human-murine chimeric IgM based on the variable regions of MAb CVV-17 was developed as previously described.^26^ CVVhIgM17 cell clones constitutively expressing the antibody with optimal expression was monitored by ELISA and IFA over 10 passages (Figure 3C–E). CVVhIgM17 had an average log_2_ EPT of 8.86, with EPTs ranging from 7.8 to 9.9 throughout the 10 passages (Figure 3C). When the EPTs of the 10 passages were analyzed using LAD regression, antibody secretion was shown to be stable with a calculated slope of 0.04 (95% CI: −0.20 to 0.26) (Figure 3D). CVVhIgM17 was purified from cell culture supernatant and tested in the MAC-ELISA with an anti-CVV-positive human serum to evaluate and compare its performance. The anti-CVV-positive human serum had a P/N value of 70.7 when diluted 1:1,000. Purified CVVhIgM17 had P/N values of 72.9, 45.6, 17.5, and 2.7 when used at concentrations of 34, 4.3, 0.26, and 0.03 ng/mL, respectively (Figure 3E).

### Analysis of MAC-ELISA testing with CVV-positive and -negative arbovirus samples.

Performance of the CVV MAC-ELISA was evaluated using seven archived samples from six individuals within the United States who were previously diagnosed with CVV infection by 90% PRNT (PRNT_90_) with reciprocal titers ranging from 10 to 1,280. Patient 1 had an anti-CVV PRNT_90_ titer of 1,280 and was positive in the MAC-ELISA with a P/N value of 46.20 and an NBR value of 161.2. Patient 4 had a PRNT_90_ titer of 160 at 17 days post-symptom onset (DPO). Another sample taken 38 DPO was tested with the paired sample in the MAC-ELISA, and both were positive (P/Ns = 63.44 and 54.73, NBRs = 124.12 and 53.40, respectively). Patients 5 and 6 had PRNT_90_ titers of 10 and 80, respectively. Patient 5’s sample was equivocal in the MAC-ELISA (P/N = 2.98, NBR = 5.15), whereas patient 6’s sample was positive (P/N = 20.78, NBR = 49.29). Patients 2 and 3 were PRNT_90_ positive with titers of 80 and 10, respectively. Patient 2’s sample was negative in the MAC-ELISA (P/N = 1.53, NBR = 4.35), whereas patient 3’s sample was positive (P/N = 15.06, NBR = 49.52) (Table 1). Taken together, the CVV MAC-ELISA was able to positively detect anti-CVV IgM in most of the samples with CVV PRNT_90_ titers >10.

**Table 1:** Archived diagnostic samples with suspected CVV infection

Patient	DPO	CVV PRNT*	MAC-ELISA		
			P/N^†^	NBR^‡^	Result^§^
1	132	1,280	46.2	161.22	POS
2	Unknown^‖^	80	1.53	4.35	NEG
3	Unknown^‖^	10	15.06	49.52	POS
4	17	160	63.44	124.12	POS
4b	38	NT	54.73	53.4	POS
5	14	10	2.98	5.15	EQ
6	188	80	20.78	49.29	POS
PHS-1^¶^	–	–	49.01	–	–
PHS-2	–	–	19.26	–	–

CVV = Cache Valley virus; DPO = days post-symptom onset; EQ = equivocal; MAC-ELISA = IgM antibody capture ELISA; NBR = nonspecific background reactivity; NEG = negative; NT = not tested; PHS-1 = positive human serum 1; PHS-2 = positive human serum 2; POS = positive ; PRNT = plaque reduction neutralization test.

* Values are the reciprocal of the sample dilution which inhibits 90% of CVV plaques.

^†^ P/N, positive-to-negative ratio, defined as the mean optical density at 450 nm (OD450) value of the sample reacted on CVV antigen divided by the mean OD450 value of the negative human serum reacted on CVV antigen.

^‡^ NBR, defined as the mean OD450 value of the sample reacted on CVV antigen divided by the mean OD450 value of the sample reacted on normal antigen.

^§^ MAC-ELISA interpretations: POS: P/N ≥3.0 and NBR ≥2.0; EQ: 2.0 ≤ P/N < 3.0 and NBR ≥2.0; NEG: P/N <2.0 and NBR any value; uninterpretable: P/N ≥2.0 and NBR <2.0.

^‖^ Unknown = sample from asymptomatic donor.

^¶^ The average P/N for PHS-1 and PHS-2 over four separate runs.

The CVV MAC-ELISA was evaluated with 44 archived samples from patients presumptively diagnosed with another arbovirus, including nine each with La Crosse virus (LACV) and Jamestown Canyon virus, four each with West Nile virus (WNV) and Powassan virus, three each with chikungunya virus and dengue virus (DENV), and two each with St. Louis encephalitis virus, Zika virus (ZIKV), Eastern equine encephalitis virus (EEEV), Colorado tick fever virus, yellow fever virus, and Heartland virus. All samples were negative in the MAC-ELISA with P/N values <2.0 (Supplemental Table 2).

### Analysis of CVV MAC-ELISA testing to evaluate samples from patients in Yucatán, Mexico, who may have had previous exposure to CVV.

Previously, in a serosurvey of patients with acute febrile illness living in Yucatán, Mexico, 18% (146 out of 823 patients) were found to have orthobunyavirus-specific antibodies detected by PRNT when tested against CVV and Kairi, Cholul, South River, Maguari, and Wyeomia viruses.^9^ Given the high seroprevalence among persons living in this area, we tested 27 samples from patients presenting to a medical clinic in Yucatán with acute febrile illness with no known etiologic agent using the CVV MAC-ELISA. The PRNT was also performed, and IC_90_ reciprocal titers to CVV were determined. Three samples tested positive in the MAC-ELISA with P/Ns of 6.0, 3.7, and 3.4 and IC_90_ titers of 112, 37, and <20 for samples H5, H9, and H10, respectively (Table 2). Because sample H10 had little or zero neutralizing antibody, the MAC-ELISA result for this sample could indicate a false positive. More samples with anti-CVV neutralizing antibody are needed to better determine a correlation between positive CVV PRNT and MAC-ELISA results. Interestingly, 40% (10 of 25) of the samples had detectable neutralizing antibodies to CVV (>20), higher than what was previously reported in the same area in 2012.^9^

**Table 2:** Samples from patients residing in the Yucatán Peninsula, Mexico, with acute febrile illness of unknown etiology

Sample	PRNT* IC_90_	MAC-ELISA	
		P/N^†^	Result^‡^
H1	<20	3.2	UI
H2	62	1.3	NEG
H3	79	1.3	NEG
H4	46	1.7	NEG
H5	112	6.0	POS
H6	NT	1.3	NEG
H7	<20	0.9	NEG
H8	<20	1.7	NEG
H9	37	3.7	POS
H10	<20	3.4	POS
H11	<20	1.6	NEG
H12	<20	1.3	NEG
H13	<20	2.5	EQ
H14	30	2.1	UI
H15	<20	3.9	UI
H16	<20	1.4	NEG
H17	29	1.9	NEG
H18	<20	2.0	EQ
H19	NT	1.0	NEG
H20	<20	1.6	NEG
H21	<20	1.1	NEG
H22	72	1.9	NEG
H23	323	3.0	EQ
H24	<20	1.3	NEG
H25	<20	1.5	NEG
H26	<20	1.9	NEG
H28	55	1.2	NEG
CVVhIgM17^§^	–	62.4	–

IC_90_ = 90% inhibitory concentration; EQ = equivocal; MAC-ELISA = IgM antibody capture ELISA; NEG = negative; NT = not tested; POS = positive; PRNT = plaque reduction neutralization test; UI, uninterpretable.

* Plaque reduction neutralization test values are the reciprocal of the sample dilution that inhibits 90% of CVV plaques calculated as IC_90._

^†^ P/N, positive-to-negative ratio, defined as the mean value of optical density at 450 nm (OD450) of the sample reacted on CVV antigen divided by the mean OD450 value of the negative human serum reacted on CVV antigen.

^‡^ MAC-ELISA interpretations: positive, P/N ≥3.0 and nonspecific background reactivity (NBR) ≥2.0; equivocal, 2.0 ≤ P/N < 3.0 and NBR ≥2.0; negative, P/N <2.0 and NBR any value; uninterpretable, P/N ≥2.0 and NBR <2.0.

^§^ CVVhIgM17 lot no. TC01980 was diluted 1:3,000 for use as the positive control in the assay, and the average P/N value was calculated over four separate plates.

## DISCUSSION

Cache Valley virus cases are rare in humans, and testing for exposure to the virus is not included in many diagnostic testing algorithms. Viremia is transient and often undetectable after symptoms become apparent, making utilization of diagnostic molecular methods difficult. In this study, we developed a CVV MAC-ELISA that is similar to those previously published and used for detection of arboviral infections.^28^^,^^30^ To alleviate the need for human positive control serum, which is rare, we engineered a surrogate human-murine IgM to accompany the assay.

The MAC-ELISA format is used extensively for routine diagnosis of arboviruses such as WNV, LACV, ZIKV, EEEV, and DENV. The platform has been described for use in diagnosis of multiple arbovirus families and remains one of the most common diagnostic tests in use because of the rapidity and sensitivity of the assay.^26^^,^^28^^,^^30^^–^^33^ IgM is an ideal analyte for detection of acute or recent convalescent-phase viral infections, because this antibody is produced early in the course of infection in immunocompetent persons, often when symptoms of disease are still present; however, the presence of antiviral IgM antibodies in a sample depends on the timing of sample collection. Paired specimens collected at acute and convalescent stages is ideal for determining the presence of anti-viral IgM antibodies and a rise in antiviral neutralizing antibody titers.

In this report, we outline the process of developing and evaluating the components of the MAC-ELISA for CVV. We found that antigen produced in Vero cells and inactivated with BPL was reactive with both murine MAbs and human immune sera. Monoclonal antibody CVV-14, reactive to the N protein of CVV, was previously shown to produce high P/N values with a CVV-immune reactive serum in comparison with the NHS control in a pilot MAC-ELISA, and therefore this MAb was conjugated to HRP for inclusion as the detector antibody in the assay.^29^

To make CVV-hIgM recombinant antibody for use as a positive control in place of positive human sera, for which availability is scarce, we evaluated six previously developed anti-CVV MAbs (CVV-4, -13, -14, -15, -17, and -18)^29^ and found that CVVhIgM17 had the highest reactivity in the MAC-ELISA. The HEK-293 cell line secreting CVVhIgM17 was shown to stably secrete antibody over 10 passages with log_2_ EPTs ranging from 7.8 to 9.9. The near-zero slope of the LAD regression line of 0.04 indicated that antibody was consistently secreted throughout the cell passages. Purified CVVhIgM17 was tested in the MAC-ELISA alongside positive human serum (PHS) and was found to react similarly, with P/N values of 72.9 when used at a concentration of 34 ng/mL compared with 70.7 when PHS was diluted to 1:1,000. Purified CVVhIgM17 was diluted as low as 0.26 ng/mL in the MAC-ELISA and still produced a P/N value of 17.5. The use of murine-human chimeric IgM antibodies for flaviviruses, alphaviruses, and the California serogroup orthobunyaviruses has increased our capacity to test for rare arboviral diseases for which PHS is limited.^26^^,^^32^^,^^33^ Using MAbs that are broadly cross-reactive among a genus or serogroup of viruses allows for one engineered control to be used in the MAC-ELISA to test for the presence of disease to several viruses. Monoclonal antibody CVV-17 was previously found to be cross-reactive with Tensaw virus, Tlacotalpan virus, Fort Sherman virus, Playas virus, and Potosi virus, making it a potential positive control in MAC-ELISAs for these related orthobunyaviruses if future needs arise.^29^

Only seven human clinical cases of CVV infections have been reported in the United States to date; of these, three patients died.^12^^–^^18^ Acquiring enough samples from patients with a recent CVV infection with anti-CVV IgM is difficult and leads to challenges when developing and validating new serological assays that detect CVV. Evaluation of a limited number of archived specimens either diagnostically confirmed CVV positive or positive for other arboviruses were tested in the CVV MAC-ELISA to begin to assess assay performance and determine whether these samples contained anti-CVV IgM. Of the seven archived samples from six patients diagnosed with CVV infection that had CVV PRNT_90_ titers >10, six were either positive or equivocal in the MAC-ELISA, demonstrating the presence of CVV IgM in these samples and suggesting initial good agreement with PRNT_90_ titer results. One sample with a PRNT_90_ titer of 80 tested negative in the MAC-ELISA; however, this sample came from an asymptomatic donor. Therefore, the positive PRNT result may indicate a previous infection, and the negative MAC-ELISA result may not be a false negative. Of the 44 archived samples from patients diagnosed with another arbovirus infections, all were negative in the CVV MAC-ELISA, suggesting initial good analytical specificity of the assay (44/44 = 100%). Further studies will need to be performed as samples are identified to fully consider aspects of CVV MAC-ELISA validation, such as diagnostic sensitivity and specificity, false-positive and -negative rates, diagnostic accuracy, and positive and negative predictive values.

Information on the seroprevalence of CVV in humans is limited, as few studies have been conducted. Rates of seroprevalence range from 3% to 18%, depending on the location of the study.^9^^–^^11^^,^^34^ Although seroprevalence of CVV infections is rare in the United States, CVV and other related orthobunyaviruses have been shown to circulate in Yucatán, Mexico, where researchers found an 18% seropositivity rate for orthobunyaviruses among febrile patients measured by PRNT with CVV and other related viruses, including Cholul, Kairi, Maguari, and Wyeomia viruses.^9^ Of these patients, 4% were specifically positive for neutralizing antibodies to CVV.^9^ Based on these data, we collected and evaluated samples from the same area from patients with an acute febrile illness suspected to be arthropod-borne with no known etiology in the MAC-ELISA and PRNT. We found 40% (10 of 25) of samples tested had anti-CVV neutralizing antibody, and of those, two were positive in the MAC-ELISA. These results may indicate previous exposure to CVV or another related orthobunyavirus that may circulate in the region in those patients; however, evidence of these infections being recent is unlikely because several had negative results in the MAC-ELISA. Also, CVV or other related orthobunyaviruses that circulate in the region may produce high seroprevalence among residents but do not cause acute disease that is easily diagnosed. It is important to note that additional clinical information such as symptom onset dates, clinical patient information, and any alternate testing, in conjunction with MAC-ELISA and differential PRNT_90_ results, is required for an accurate diagnosis and final CVV case classification. In addition, persistence of anti-CVV IgM after infection is unknown and may confound future diagnostic testing. More studies should be conducted to understand antibody kinetics after CVV infection.

Cache Valley virus is a One Health concern, because of its ability to cause severe disease in ruminants, including congenital disease, and in humans; however, teratogenicity in humans in unknown and understudied. The MAC-ELISA described here will expand diagnostic testing for CVV and help to identify CVV human infections, including less severe cases of human disease, which may be underrecognized.^13^ The MAC-ELISA has the potential to help in our understanding of the risk for teratogenicity in humans by determining a correlation between human congenital disease and presence of anti-CVV IgM in future seroprevalence studies. The assay could also be used as a veterinary diagnostic tool with the inclusion of appropriate controls. Implementation of the CVV MAC-ELISA will ultimately lead to a better understanding of the spectrum of disease caused by CVV.

## Supplemental Materials

10.4269/ajtmh.24-0360 Supplemental Materials
