# Supplementary material for: Development of a Diagnostic IgM Antibody Capture ELISA for Detection of Anti-Cache Valley Virus Human IgM
Source: Am J Trop Med Hyg. 2024 Nov 19;112(2):386–95. doi: 10.4269/ajtmh.24-0360 (PMC11803673; doi:10.4269/ajtmh.24-0360)
Supplement: Supplemental Materials [file tpmd240360.SD1.pdf]

Supplemental Table 1. Primers for cloning the VH and Vκ regions of MAbs CVV-14, CVV-15, and CVV-17 into pVITRO-Trastuzumab-IgM/κ for expression in HEK-293 cells.

**Primer**	**Sequences 5' to 3'**
CVV14_HvF	GCTAATTCAAAGCAACCGGTatggacaggcttacttcttc
CVV14_IgK_R	gaagaagtaagcctgtccatACCGGTTGCTTTGAATTAGC
CVV14_IgH_F	cacggtcaccgtctcctcagGATCTGCATCCGCTCCAACC
CVV14_HvR	GGTTGGAGCGGATGCAGATCctgaggagacggtgaccgtg
CVV14_KvF	CTAATTCAAAGCAATCCGGAatggattttcaagtgcagat
CVV14_IgH_R	atctgcacttgaaaatccatTCCGGATTGCTTTGAATTAG
CVV14_IgK_F	gaccaagctggaggtgaaacGTACGGTGGCGGCGCCATCT
CVV14_KvR	AGATGGCGCCGCCACCGTACgtttcacctccagcttggtc
CVV15_KvF	CTAATTCAAAGCAATCCGGAatgatgtcctctgctcagtt
CVV15_IgH_R	aactgagcagaggacatcatTCCGGATTGCTTTGAATTAG
CVV15_IgK_F	caccaagctggaaatcaaacGTACGGTGGCGGCGCCATCT
CVV15_KvR	AGATGGCGCCGCCACCGTACgtttgatttccagcttggtg
CVV15_HvF	GCTAATTCAAAGCAACCGGTatggaaaggcactggatctt
CVV15_IgK_R	aagatccagtgcctttccatACCGGTTGCTTTGAATTAGC
CVV15_IgH_F	tctggtcaccgtctctgcagGATCTGCATCCGCTCCAACC
CVV15_HvR	GGTTGGAGCGGATGCAGATCctgcagagacggtgaccaga
CVV17_KvF	CTAATTCAAAGCAATCCGGAatggattttcaagtgcagct
CVV17_IgH_R	agctgcacttgaaaatccatTCCGGATTGCTTTGAATTAG
CVV17_IgK_F	gaccaagctggagctgaaacGTACGGTGGCGGCGCCATCT
CVV17_KvR	AGATGGCGCCGCCACCGTACgtttcagctccagcttggtc
CVV17_HvF	GCTAATTCAAAGCAACCGGTatggacaggcttacttcttc
CVV17_IgK_R	gaagaagtaagcctgtccatACCGGTTGCTTTGAATTAGC
CVV17_IgH_F	cacggtcaccgtctcctcagGATCTGCATCCGCTCCAACC
CVV17_HvR	GGTTGGAGCGGATGCAGATCctgaggagacggtgaccgtg

Supplemental Table 2. Evaluation of the CVV MAC-ELISA when tested with archived human diagnostic samples presumptively positive to another arbovirus.

Sample	MAC-ELISA P/N[a]	MAC-ELISA Result[b]
LACV[c]-1	0.5	NEG
LACV-2	0.6	NEG
LACV-3	1.1	NEG
LACV-4	0.6	NEG
LACV-5	1.2	NEG
LACV-6	1.3	NEG
LACV-7	0.7	NEG
LACV-8	0.7	NEG
LACV-9	1.1	NEG
JCV-1	0.6	NEG
JCV-2	0.4	NEG
JCV-3	1.3	NEG
JCV-4	0.4	NEG
JCV-5	0.6	NEG
JCV-6	0.7	NEG
JCV-7	0.8	NEG
JCV-8	0.4	NEG
JCV-9	0.8	NEG
SLEV-1	0.5	NEG
SLEV-2	0.9	NEG
WNV-1	0.6	NEG
WNV-2	0.7	NEG
WNV-3	1.7	NEG
WNV-4	0.6	NEG
ZIKV-1	0.7	NEG
ZIKV-2	0.9	NEG
POWV-1	0.5	NEG
POWV-2	1.7	NEG
POWV-3	0.8	NEG
POWV-4	2.0	NEG
CHIKV-1	0.8	NEG
CHIKV-2	0.3	NEG
CHIKV-3	1.1	NEG
EEEV-1	0.3	NEG
EEEV-2	0.8	NEG
DENV-1	0.5	NEG
DENV-2	0.5	NEG

DENV-3	0.6	NEG
CTFV-1	0.5	NEG
CTFV-2	0.5	NEG
YFV-1	0.4	NEG
YFV-2	0.6	NEG
HTRV-1	0.8	NEG
HTRV-2	1.2	NEG
PHS-2*	14.8	

[a]P/N, positive to negative ratio, defined as the mean OD450 value of the sample reacted on CVV antigen divided by the mean OD450 value of the negative human serum reacted on CVV antigen

[b] NBR, nonspecific background reactivity, defined as the mean OD450 value of the sample reacted on CVV antigen divided by the mean OD450 value of the sample reacted on normal antigen

[c]MAC-ELISA interpretations: Positive: P/N ≥ 3.0 and NBR ≥ 2.0; Equivocal: 2.0 ≤ P/N < 3.0 and NBR ≥ 2.0; Negative: P/N < 2.0 and NBR any value; Uninterpretable: P/N ≥ 2.0 and NBR < 2.0.

[d]Archived samples from patients presumptively diagnosed with an arbovirus not related to CVV were tested in the MAC-ELISA. LACV = La Crosse virus, JCV= Jamestown Canyon virus, SLEV = St. Louis encephalitis virus, WNV = West Nile virus, ZIKV = Zika virus, POWV = Powassan virus, CHIKV = chikungunya virus, EEEV = Eastern equine encephalitis virus, DENV = dengue virus, CTFV = Colorado tick fever virus, YFV = yellow fever virus, HRTV = Heartland virus.

*PHS-2 was diluted 1:400 for use as the positive control in the assay and the average P/N value was calculated over six separate plates.
